# Cognitive and Psychiatric Effects of STN versus GPi Deep Brain Stimulation in Parkinson's Disease: A Meta-Analysis of Randomized Controlled Trials

**DOI:** 10.1371/journal.pone.0156721

**Published:** 2016-06-01

**Authors:** Jia-Wei Wang, Yu-Qing Zhang, Xiao-Hua Zhang, Yun-Peng Wang, Ji-Ping Li, Yong-Jie Li

**Affiliations:** Beijing Institute of Functional Neurosurgery, Department of Functional Neurosurgery, Xuanwu Hospital, Capital Medical University, Beijing, P.R. China; University of Medicine & Dentistry of NJ—New Jersey Medical School, UNITED STATES

## Abstract

**Background:**

Deep brain stimulation (DBS) of either the subthalamic nucleus (STN) or the globus pallidus interna (GPi) can reduce motor symptoms in patients with Parkinson’s disease (PD) and improve their quality of life. However, the effects of STN DBS and GPi DBS on cognitive functions and their psychiatric effects remain controversial. The present meta-analysis was therefore performed to clarify these issues.

**Methods:**

We searched the PUBMED, EMBASE, and the Cochrane Central Register of Controlled Trials databases. Other sources, including internet-based clinical trial registries and grey literature sources, were also searched. After searching the literature, two investigators independently performed literature screens to assess the quality of the included trials and to extract the data. The outcomes included the effects of STN DBS and GPi DBS on multiple cognitive domains, depression, anxiety, and quality of life.

**Results:**

Seven articles related to four randomized controlled trials that included 521 participants were incorporated into the present meta-analysis. Compared with GPi DBS, STN DBS was associated with declines in selected cognitive domains after surgery, including attention, working memory and processing speed, phonemic fluency, learning and memory, and global cognition. However, there were no significant differences in terms of quality of life or psychiatric effects, such as depression and anxiety, between the two groups.

**Conclusions:**

A selective decline in frontal-subcortical cognitive functions is observed after STN DBS in comparison with GPi DBS, which should not be ignored in the target selection for DBS treatment in PD patients. In addition, compared to GPi DBS, STN DBS does not affect depression, anxiety, and quality of life.

## Introduction

As a leading cause of morbidity and mortality worldwide, Parkinson’s disease (PD) has received considerable time and attention in the clinic [1]. It has been reported that the prevalence of PD varies from 41 to 1903 cases per 100,000 people annually, with its incidence increasing steadily with age [2], and with many PD patients suffering permanent disability or even death [3,4]. Unfortunately, although intensive research has been performed in studies of PD, there is currently no cure for PD, and therapeutic interventions for PD remain limited [5–7].

The gold standard treatment for the management of PD consists of antiparkinsonian medication and surgical interventions [8]. Generally, antiparkinsonian medication is mainly administered to PD patients in the early stage of the disease following an initial diagnosis[9], while surgery using deep brain stimulation (DBS) tends to be performed on those in the advanced stage of the disease who have experienced drug-induced complications or reduced responsiveness to the best medical therapies [10]. A wealth of evidence from randomized controlled trials (RCTs) have confirmed that administering DBS plus medication results in significant improvements in motor symptoms and quality of life in PD patients in comparison to administering medication alone[11–14]. These results are consistent with the results of a recently performed systematic review[15]. The subthalamic nucleus (STN) and globus pallidus interna (GPi) are the two most commonly selected targets that have been extensively explored in the clinic. RCTs comparing STN DBS and GPi DBS have indicated that patients with PD experience similar improvement in motor function after either pallidal or subthalamic stimulation [16–18]. However, the effect of STN DBS and GPi DBS on non-motor symptoms, especially cognition, psychiatric symptoms and quality of life, are controversial [17–21]. Because cognitive and psychiatric symptoms have important effects on the quality of life in PD patients, it is necessary to clarify the roles of both neurostimulatory models on cognitive and psychiatric effects to improve rational management and outcomes.

To our knowledge, there are some meta-analyses reviewing the effects of both STN DBS and GPi DBS on cognitive and psychiatric effects[22–25]. However, some limitations are noted in these existing literatures. For example, as described in the text by the Combs and co-authors, RCTs are considered the “gold standard” for evaluating efficacy of medical interventions[22]. However, only one RCT study (COMPARE trial)[17,26] comparing two targets was included in their meta-analysis while the remainder of included studies mainly investigated the outcomes before and after each DBS treatment [22]. Similar situation is found in the study by Couto and co-authors [25]. In addition, the neuropsychological outcome after DBS from the newly published NSTAPS study[21] was not included in all the previous meta-analyses [22–25]. These issues highlight the need to re-appraise the current evidence. Thus, the present meta-analysis was performed to compare STN DBS with GPi DBS in patients with PD to investigate postoperative changes in cognitive function and psychiatric symptoms as well as to assess their effects on quality of life.

## Materials and Methods

### Study identification

A systematic review of the published literature was performed to identify all of the clinical RCTs in which STN DBS was compared to GPi DBS in patients with PD. All of the included RCT studies contained definite inclusion criteria and at least one standardized neuropsychological or cognitive instrument must have been presented before and after DBS surgery. Studies that were either not RCTs or did not directly involve an evaluation of the cognitive and psychiatric effects of STN DBS and GPi DBS in PD patients were eliminated.

### Search strategy

We (Wang J-W and Zhang Y-Q) performed an electronic search for relevant articles in three databases, including PUBMED (up to July 2015), EMBASE (up to July 2015) and the Cochrane Central Register of Controlled Trials (CENTRAL, up to July 2015), without language limitations based on text words or MeSH terms such as “deep brain stimulation”, “neurostimulation”, “cognition”, “neuropsychology”, “psychiatry”, and “Parkinson disease”. Moreover, the grey literature in the OpenGrey database (a System for Information on Grey Literature in Europe) and the National Technical Information Service (NTIS) were also searched. In addition, internet-based clinical trial registries, such as ClinicalTrials.gov, International Clinical Trials Registry Platform (ICTRP) and International Standard Randomized Controlled Trial Number Register (ISRCTN), were also searched. Finally, the Related Articles function on PUBMED was used, and the reference lists of relevant articles were reviewed. For full details of the search strategy used in this study, please see S1 Search Strategy. The search was performed independently by two investigators and was completed in July 2015.

### Literature screening

Two investigators (Wang Y-P and Zhang X-H) first reviewed the titles and abstracts of the identified studies and then excluded those that were either obviously irrelevant or duplicates. The full text of each of the remaining studies was then independently reviewed to determine its eligibility. A third investigator (Li Y-J) as included if there was a disagreement. When necessary, we contacted the research authors for further information.

### Quality assessment

The quality of the eligible studies was formally evaluated using the Cochrane Collaboration’s tool for assessing the risk of bias in randomized trials (version 5.1.0). Specifically, the studies were judged using the following terms: random sequence generation, allocation sequence concealment, blinding of participants and personnel, blinding of outcome assessments, incomplete outcome data, selective outcome reporting, and other potential threats to validity. The risk of bias for each item was categorized as high, unclear or low.

### Data extraction

The following data were extracted from each study: baseline characteristics, design and objective, number of patients, timing of measurements, the main results of the study, and follow-up results. In the present study, a battery of neurocognitive tests were categorized into the following six neuropsychological domains: global cognitive functioning, attention, working memory and processing speed, executive functioning, verbal fluency, language, as well as learning and memory. Although most neuropsychological measures assess multiple cognitive functions, each test was, consistent with accepted clinical practice, assigned to the one domain whose integrity the measure is thought to predominantly reflect [27,28]. The outcomes included the effects of STN DBS or GPi DBS on global cognitive functions, attention, working memory and processing speed, executive functions, verbal fluency, language, learning and memory, depression, anxiety, and quality of life. In situations when two or more studies were drawn from the same RCT, the data from the last follow-up was used.

### Statistical analysis

A meta-analysis of prospective RCTs was performed using the heterogeneity method in Review Manager for Windows (version 5.3, Cochrane Collaboration and Update Software). Standard Cochran Q and I^2^ statistics were used to assess the heterogeneity between studies, and they were pre-specified as P<0.10 or I^2^>50% in the present study. The standardized mean difference (SMD) was used as the effect parameter for the meta-analysis when the analyzed domains involved multiple testing instruments. Weighted mean difference (WMD) was used when studies used the same instrument to assess the domain. In addition, it should be noted that the SMD method does not correct for differences in the direction of the scale. If some scales increased with disease severity while others decreased, it was essential to multiply the mean values from one set of studies by –1 to ensure that all of the scales pointed in the same direction (Cochrane Handbook for Systematic Reviews of Interventions, version 5.1.0, Chapter 9.2.3.2). A 95% confidence interval (CI) was used to interpret the results. A fixed-effects model was used to merge the values of the SMD or WMD to estimate the overall effect size when heterogeneity between studies could not be obtained. Otherwise, a random-effects model was used in the statistical analysis. All of the tests were 2-sided, and statistical significance was defined as a probability value of <0.05 unless specifically stated.

## Results

### Characteristics of included studies

**Fig 1:**
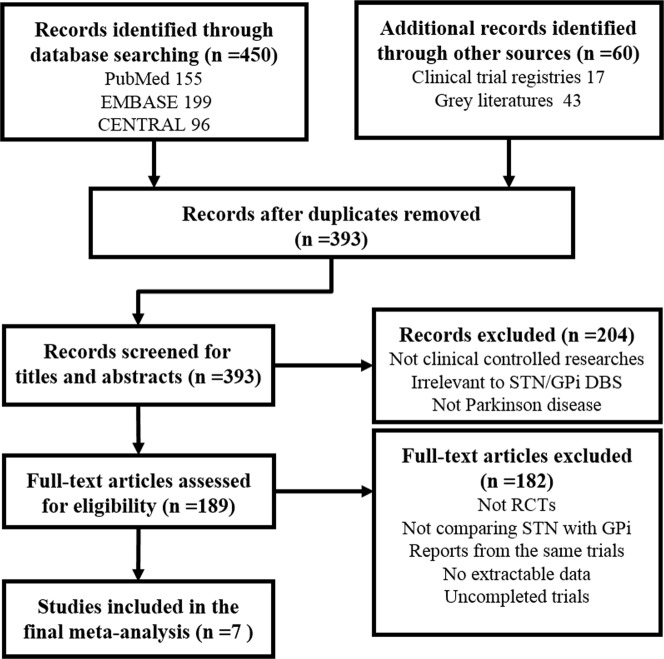
Flow chart used to include studies in the present meta-analysis.

In total, 510 articles were initially identified, and 503 articles were excluded, leaving 7 articles for the final analysis. Fig 1 shows the flow chart used to obtain the search results and the screening process. The PRISMA checklist is listed in S1 PRISMA Checklist.

Table 1 summarizes the demographic data in the seven included articles. As shown in Table 1, the seven included articles involved four prospective RCTs, including the Rothlind study [28], the COMPARE trial [17,26], the CSP468 study [16,29], and the NSTAPS study [21,30]. A total of 521 participants were enrolled in the four RCTs. Of these, 255 (48.9%) PD patients included in the group received STN DBS treatment, and 405 (77.7%) of the PD patients were male. All of the included trials incorporated distinct inclusion criteria (S1 Table). Each trial described the baseline characteristics of the enrolled participants. There were no significant differences in the baseline characteristics of participants between the groups in these trials.

**Table 1:** Baseline characteristics of the included trials.

Author & Year	Groups	No.	Age (years)	Gender (M/F)	Disease duration (years)	Hoehn-Yahr stage off-medication	Mattis dementia rating scale	Microelectrode confirmation	Placement verified	Stimulation parameters	Assessment time point
**Rothlind et al, 2007 (Rothlind**	GPi	23	60.2±8.83	18/5	13.3±6.4	3.3±0.56	139.4±3.98	Yes	Yes	185Hz, 92.3μs, 3.3 V 185Hz, 95μs, 3.5 V*	Base, 15 months
**study)**	STN	19	61.4±10.11	15/4	12.9±4.3	3.3±0.45	140.4±2.87			185Hz, 60μs, 2.6 V 182.9Hz, 65.6μs, 2.6 V	
**Okun et al, 2009 and Zahodne et al, 2009**	GPi	26	60.2±6.2	17/9	12.5±3.6	2.7±0.82	138.8±4.4	Yes	Yes	151.5Hz, 84.7μs, 2.9 V	Base, 7 months
**(COMPARE Trial)**	STN	26	59.8±10	18/8	13.3±4.0	3.0±0.63	136.5±7.0			141.1Hz, 94.0μs, 2.4 V	
**Follett et al, 2010 and Weaver et al,**	GPi	152	61.8±8.7	133/19	11.5±5.4	3.3±0.9	137.5±4.8	Yes	N/A	168Hz, 95.7μs, 3.95 V	Base, 24, 36 months
**2012 (CSP 468 study)**	STN	147	61.9±8.7	116/31	11.1±5.0	3.4±0.9	137.2±5.1			165Hz, 75.9μs, 3.16 V	
**Odekerken et al, 2013 and Odekerken et al,**	GPi	65	59.1±7.8	44/21	10.8±4.2	N/A	138.7±4.0	Yes	N/A	137.5Hz, 73μs, 2.9 V	Base, 12 months
**2015 (NSTAPS study)**	STN	63	60.9±7.6	44/19	12.0±5.3		138.1±5.1			135Hz, 63.9μs, 2.6 V	

Data is shown as the mean ± SD or number. Abbreviation: N/A: not available, No.: number of patients, M/F: male/female, STN: subthalamic nucleus, GPi: globus pallidus interna

* the bilateral stimulation parameters were reported, respectively, in the study.

Among the four trials, the implantation of DBS electrodes in PD patients was bilateral in three trials (the Rothlind [28], NSTAPS [21,30] and CSP468 studies [16,29]) and unilateral in one trial (COMPARE trial [17,26]). The mean DBS stimulation parameter was not compared in the Rothlind study, while three other studies indicated that GPi DBS required significant increases in the stimulation amplitude in PD patients in terms of voltage, frequency or pulse width compared to STN DBS. In addition, the maximal duration of the follow-up period ranged from seven months to three years.

### Risk of bias in the included studies

**Fig 2:**
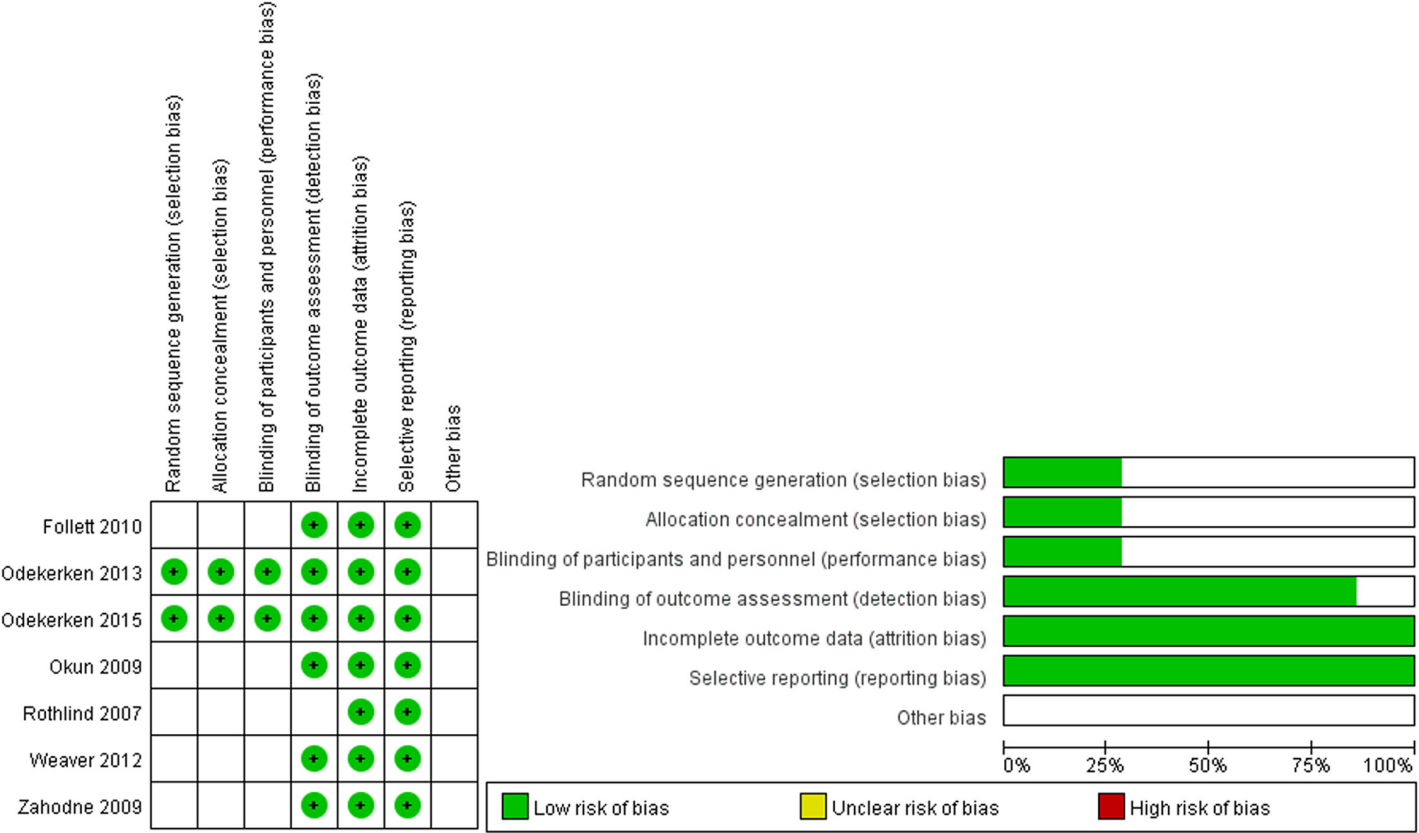
Risk-of-bias assessment of the included trials.

As described in Fig 2, the risk of bias within each study for every item was graded. Although all four of the included trials used a statement such as “randomly”, “randomization” or “randomized” in its Methods section, only one trial (NSTAPS) reported its full methodology for implementing random sequence generation and allocation concealment in detail[21,30]. The NSTAPS trial also reported that participants and personnel were blinded despite the difficulty of doing so in the studies using surgical interventions. Moreover, there was a low risk in all of the studies except the Rothlind study [28], with regard to blinding during outcome assessments. The data analyses were based on the intention-to-treat principle in two studies (the NSTAPS study [21,30] and the CSP468 study [16,29]). In addition, the risk of attrition and reporting bias was considered to be low in all four of the included trials.

### Effects of STN DBS vs. GPi DBS on global cognition

As shown in Table 2, only one study (the CSP468 trial) explored the effects of STN DBS vs. GPi DBS on global cognition as assessed using the Mattis dementia rating scale (MDRS). The pooled WMD of global cognitive functioning at the end of the follow-up period in STN DBS compared to GPi DBS was -4.30 (95% CI: -7.98–-0.62, P = 0.02), indicating that STN DBS in PD patients may result in a lower global cognition score than GPi DBS (Table 3).

**Table 2:** Tests included in each neuropsychological domain.

	Test	GPi	STN	Studies	K
**Global cognitive**	Mattis dementia rating scale total^#^	86	66	C	1
**functioning**					
**Attention, working**	Digit span forward^#^	14	15	A	1
**memory and**	Digit span backward^#^	14	15	A	1
**processing speed**	WAIS-arithmetic^#^	14	15	A	1
	WAIS -processing speed index^#^	84	64	C	1
	WAIS -working memory index^#^	84	63	C	1
	WAIS -letters and numbers^#^	58	56	D	1
	WAIS -digit span^#^	58	56	D	1
	Trail making test part A*	72	71	A, D	2
	WAIS -Digit symbol^#^	14	15	A	1
	Stroop word reading*	72	71	A, D	2
	Stroop color naming*	72	71	A, D	2
	Vienna test system1-reaction*	58	56	D	1
	Vienna test system1-motoric*	58	56	D	1
	Vienna test system3-reaction*	58	56	D	1
	Vienna test system3-motoric*	58	56	D	1
**Executive**	Stroop color-word interference*	224	218	A, C, D	3
**functioning**	Trial making test part B*	72	71	A, D	2
	Wisconsin card sorting test completed categories^#^	58	56	D	1
	Wisconsin card sorting test No. of errors*	58	56	D	1
	Wisconsin card sorting test perseverative response^#^	81	63	C	1
	Wisconsin card sorting test perseverative error*	58	56	D	1
**Verbal fluency**	Phonemic(letter) fluency^#^	180	157	A,B,C,D	4
	Semantic(category) fluency^#^	180	157	A,B,C,D	4
**Language**	Boston naming test^#^	157	134	A, C, D	3
	BDAE-CIM^#^	14	15	A	1
	WAIS -similarity^#^	58	56	D	1
**Learning and**	Brief visuospatial memory test total^#^	99	80	A, C	2
**memory**	Brief visuospatial memory test delayed recall^#^	99	80	A, C	2
	RAVLT total and immediate recall ^#^	72	71	A, D	2
	RAVLT delayed recall^#^	72	71	A, D	2
	Hopkins verbal learning test total^#^	85	64	C	1
	Hopkins verbal learning test delayed recall^#^	85	64	C	1
	RBMT total^#^	58	56	D	1
	RBMT immediate recall^#^	58	56	D	1
	RBMT delayed recall^#^	58	56	D	1
**Depression**	Beck depression inventory*	121	99	A, B, C	3
**Anxiety**	State trait anxiety inventory-State*	36	36	A, B	2
	State trait anxiety inventory-Trait*	36	36	A, B	2
**Quality of Life**	Parkinson’s disease questionnaire-39 total*	103	82	B, C	2
	Parkinson’s disease quality of life questionnaire^#^	62	63	D	1

Data indicates the number of patients in each group.

Studies column indicates the specific studies using the cognitive test. A: Rothlind study, B: COMPARE trial, C: CSP468 study, D: NSTAPS study.

K = the number of studies evaluating each domain.

Abbreviations: BDAE-CIM: Boston diagnostic aphasia examination-complex ideational material, RAVLT: Rey auditory verbal learning test, RBMT: Rivermead behavioral memory test, WAIS: Wechsler adult intelligence scale.

# A higher score indicates better functioning.

* A higher score indicates worse functioning.

**Table 3:** Results for the neuropsychological domains.

	Heterogeneity	SMD[95%CI]	WMD[95%CI]
**Global cognitive functioning**	—	—	-4.30[-7.98, -0.62]*
**Attention, working memory and processing speed**	Chi² = 14.22, df = 17 (P = 0.65); I² = 0%	-0.21 [-0.31, -0.10] *	—
**Executive functioning**	Chi² = 15.30, df = 8 (P = 0.05); I² = 48%	-0.12 [-0.30, 0.06]	—
**Verbal fluency**	Chi² = 6.45, df = 7 (P = 0.49);	-0.24 [-0.39, -0.09] *	—
**Phonemic fluency**	I² = 0% Chi² = 4.13, df = 3 (P = 0.25);	—	-2.93 [-5.41, -0.44] *
**Semantic fluency**	I² = 27%Chi² = 3.47, df = 3 (P = 0.33); I² = 13%	—	-1.55 [-3.14, 0.04]
**Language**	Chi² = 2.62, df = 4 (P = 0.62); I² = 0%	0.05 [-0.14, 0.24]	—
**Learning and memory**	Chi² = 11.68, df = 12 (P = 0.47); I² = 0%	-0.16 [-0.27, -0.05] *	—
**Depression**	Chi² = 0.68, df = 2 (P = 0.71); I² = 0%	—	1.37 [-0.57, 3.30]
**Anxiety State anxiety Trait anxiety**	Chi² = 1.88, df = 3 (P = 0.60);	-0.02 [-0.35, 0.31]	—
	I² = 0% Chi² = 0.58, df = 1 (P = 0.45);	—	-1.85 [-7.72, 4.02]
	I² = 0% Chi² = 0.69, df = 1 (P = 0.41); I² = 0%	—	1.51 [-3.22, 6.25]
**Quality of Life**	Chi² = 7.43, df = 2 (P = 0.02); I² = 73%	-0.15 [-0.61, 0.31]	—

Abbreviation: SMD: standardized mean difference, WMD: weighted mean difference

* P <0.05.

### Effects of STN DBS vs. GPi DBS on attention, working memory and processing speed

In the three included trials (the Rothlind study, CSP468 study, and NSTAPS study), multiple neurocognitive tests were used to investigate the effects of STN DBS vs. GPi DBS on attention, working memory and processing speed (Table 2). Our results show that STN DBS resulted in a significantly decreased score in the cognitive domain of attention, working memory and processing speed compared to GPi DBS (SMD: -0.21, 95% CI: -0.31–-0.10, P < 0.0001), which was associated with statistically non-significant heterogeneity (P = 0.65, I^2^ = 0%) (Table 3).

### Effects of STN DBS vs. GPi DBS on executive functioning

Data about the effects of STN DBS vs. GPi DBS on executive functioning were reported in the Rothlind study, CSP468 study, and NSTAPS study (Table 2). As described in Table 3, the pooled SMD of executive functioning at the end of follow-up in patients who received STN DBS compared to those who received GPi DBS was -0.12 (95% CI: -0.30–0.06, P = 0.19) and was associated with statistically significant heterogeneity (P = 0.05, I^2^ = 48%), indicating that STN DBS in PD patients did not result in a significant decline in executive functioning in comparison to GPi DBS.

### Effects of STN DBS vs. GPi DBS on verbal fluency

All four of the included trials reported on the effects of STN DBS vs. GPi DBS on verbal fluency (Table 2). Two components of verbal fluency, phonemic fluency and semantic fluency, were differentially affected by PD [31]. The data on verbal fluency was initially analyzed as a cognitive domain (phonemic fluency and semantic fluency together) and then disaggregated into individual phonemic fluency and semantic fluency components to better explain the variance in terms of separate groups.

As shown in Table 3, the pooled SMD for verbal fluency at the end of the follow-up period for STN DBS compared to GPi DBS was -0.24 (95% CI: -0.39–-0.09, P = 0.002) and was associated with statistically non-significant heterogeneity (P = 0.49, I^2^ = 0%). Further subgroup analysis, based on the type of verbal fluency, indicated that between the STN DBS and GPi DBS groups, only the difference in phonemic fluency was significant (MD: -2.93, 95% CI: -5.41–-0.44, P = 0.02), while there was no remarkable difference in terms of semantic fluency between the two groups (MD: -1.55, 95% CI: -3.14–0.04, P = 0.06). In addition, both of subgroup analyses exhibited statistically non-significant heterogeneity.

### Effects of STN DBS vs. GPi DBS on language

Data about the effects of STN DBS vs. GPi DBS on language functions were reported in three studies other than the COMPARE trial (Table 2). As described in Table 3, the pooled SMD for language at the end of the follow-up period using STN DBS compared to GPi DBS was 0.05 (95% CI: -0.14–0.24, P = 0.63) and was associated with statistically non-significant heterogeneity (P = 0.62, I^2^ = 0%), indicating that STN DBS in PD patients showed similar effects on language function to the effects of GPi DBS.

### Effects of STN vs. GPi DBS on learning and memory

As shown in Table 2, three trials (the Rothlind study, CSP468 study, and NSTAPS study) explored the effects of STN DBS vs. GPi DBS on learning and memory, as assessed using multiple neurocognitive tests. The data in Table 3 indicate that the pooled SMD for learning and memory at the end of follow-up using STN DBS compared to GPi DBS was -0.16 (95% CI: -0.27–-0.05, P = 0.005) and was associated with statistically non-significant heterogeneity (P = 0.47, I^2^ = 0%), suggesting that STN DBS in PD patients might be accompanied by a stronger decline in learning and memory than that observed following GPi DBS.

### Effects of STN DBS vs. GPi DBS on depression

Data about the effects of STN DBS vs. GPi DBS on depression were reported in three studies other than the NSTAPS trial, all of which used the Beck depression inventory as the assessment scale (Table 2). As described in Table 3, the pooled WMD for depression at the end of follow-up using STN DBS compared to GPi DBS was 1.37 (95% CI: -0.57–3.30, P = 0.17) and was associated with statistically non-significant heterogeneity (P = 0.71, I^2^ = 0%), indicating that STN DBS in PD patients had similar effects on depression to those of GPi DBS.

### Effects of STN vs. GPi DBS on anxiety

As shown in Table 2, the Rothlind trial and the COMPARE trial studied the effects of STN DBS vs. GPi DBS on anxiety as assessed using the StateTrait anxiety inventory. The data in Table 3 indicate that the pooled SMD for anxiety at the end of the follow-up period using STN DBS compared to GPi DBS was -0.02 (95% CI: -0.35–0.31, P = 0.92) and was associated with statistically non-significant heterogeneity (P = 0.60, I^2^ = 0%). Further subgroup analysis based on the type of anxiety was performed and did not change the outcome of the studies in either the state anxiety or the trait anxiety subgroups, suggesting that STN DBS in PD patients had similar effects on anxiety to those of GPi DBS.

### Effects of STN vs. GPi DBS on quality of life

Data describing the effects of STN DBS vs. GPi DBS on quality of life was reported in three trials other than the Rothlind study (Table 2). As described in Table 3, the pooled SMD for quality of life at the end of the follow-up period using STN DBS compared to GPi DBS was -0.15 (95% CI: -0.61–0.31, P = 0.52) and was associated with statistically significant heterogeneity (P = 0.02, I^2^ = 73%), suggesting that there was no significant difference in quality of life in PD patients between those who received STN DBS and GPi DBS.

## Discussion

In the present meta-analysis of seven articles in four RCTs, we investigated the effects of STN DBS vs. the effects of GPi DBS on cognitive function and psychiatric symptoms and assessed their effects on quality of life in the PD patients. The main findings are as follows. (1) Compared to GPi DBS, STN DBS was associated with a decline in selected cognitive domains, including global cognition, attention, working memory and processing speed, phonemic fluency, as well as learning and memory, in PD patients after surgery. (2) There were no significant differences in terms of quality of life and psychiatric effects such as depression and anxiety between the STN DBS and GPi DBS groups.

DBS is a well-recognized treatment that is widely used in PD patients who have experienced drug-induced complications or reduced responsiveness to the best medical therapies. However, some issues, such as target selection, warrant further study. In a clinical setting, STN and GPi are the two most commonly selected targets for the DBS procedure. There are long-standing concerns about whether the best target in a PD patient is the STN or GPi. The first study that retrospectively compared STN DBS to GPi DBS was completed in 1998[32], and it found that STN DBS resulted in both greater improvements to akinesia and larger reductions in dopaminergic treatment compared with GPi DBS. This finding, together with subsequent case series that reported that the initial efficacy of GPi DBS wanes over the 1–5 years after surgery [33], led to STN DBS being the prevailing treatment for PD patients [34]. However, further RCTs have indicated that STN DBS does not result in significantly increased improvements in motor symptoms and that it may be accompanied by cognitive and behavioral complications [18]. This has triggered renewed interest in GPi DBS. To date, there is a consensus that both STN DBS and GPi DBS are effective in addressing the motor symptoms of PD and the non-motor outcomes such as cognition and behavioral outcomes also play an important role in the target selection [35]. Recently, several important studies comparing the effects of STN DBS and GPi DBS on non-motor symptoms, including the COMPARE trial [17,26], NSTAPS trial [21,30], and CSP468 study [16,29], have been completed. These issues make it possible and necessary to re-evaluate the effects of STN DBS on cognition and psychiatric changes in PD patients on the basis of current evidence. Thus, our study provides timely and substantial evidence that will be useful to clinicians during the selection of appropriate treatment strategies.

In the present meta-analysis, global cognitive function after DBS surgery, was reported in only one out of four of the included RCTs [16,29]. The CSP 468 study group investigated the effects of STN DBS and GPi DBS on global cognitive function using MDRS scores for up to thirty-six months after surgery. They found that MDRS scores showed a gradual reduction in both the STN and the GPi group over time and that the difference in MDRS scores between two groups was significant at thirty-six months post-DBS (P = 0.01). However, considering that PD patients in the STN DBS group performed slightly worse on average than patients in the GPi DBS group in some baseline neurocognitive tests and that these covariates were not adjusted for the subsequent analysis [16,29], the findings in the CSP 468 trial must be interpreted with care. Moreover, the results from previous RCTs comparing STN DBS to the best medical therapy (BMT) indicated that there was no significant difference in MDRS total scores at follow-up between the STN DBS and the BMT groups (139.6±3.8 and 140.0±3.5, respectively, P = 0.25)[36]. In addition, the evidence from another meta-analysis that assessed global cognition before and after STN DBS also showed that STN DBS did not change global cognitive ability after adjusting for heterogeneity of variance in the study effect size[27]. Furthermore, an epidemiological study of PD indicated that the point prevalence estimates for dementia in PD patients ranges from 19.7 to 35.3%[37], which is in line with a previous study that suggested that 24.5% of PD patients with STN DBS converted to dementia during the 3 years following surgery[38]. All of these issues indicate that the gradual decline observed in global cognitive function during PD may reflect the underlying progression of the disease rather than target differences.

Our results demonstrate that while the neuropsychological effects of DBS surgery are generally comparable for the two brain targets, there are also differences in the domains of attention, working memory, processing speed, verbal fluency, and learning and memory. Previous study by Perestelo-Perez et al explored the neurocognitive and psychiatric effects of DBS in comparison with pharmacological treatment[15]. And they found the figures ranging from nearly no decline in memory and 0.15 SMD decline in global cognition (dementia rating) up to 0.56 SMD decline in phonemic fluency. Our findings are roughly in the same order of magnitude. Moreover, another meta-analysis by Muslimovic et al examined the magnitude of decline across multiple cognitive domains associated with disease progression in initially non-demented, non-DBS treated, mid-stage PD patients, which demonstrated that cognitive decline in the order of up to 0.3–0.4 effect size in several domains over a mean follow-up interval of 29 months[39]. Thus, the ‘Perestelo effects’ probably adds up to the ‘Muslimovic effects’, which, at least in part, explains why STN DBS results in our study probably come on top of both of these types of decline. However, the clinical relevance of these differences is unknown, because neither our study nor previous reports have shown that functional outcome scores [16,17,23] and quality of life scores [29,30] differ between the two groups.

Currently, the mechanisms underlying the observed differences in selective cognitive domains remain unknown. Two potential factors may be responsible for these differences. Firstly, the most common and consistent cognitive side-effect after DBS[34] is a worsening in verbal fluency. However, it seems to be related to the surgical procedure itself rather than the stimulation because no substantial differences have been found between the results of on stimulation and off stimulation[17,34,40]. This indicates that some of the factors involved in DBS surgery, such as lead location and trajectory, may play roles in neurocognitive outcomes. Recently, Witt K et al explored the impact of varying cortical lead entry point, subcortical electrode path, and the position of the active electrode contacts on neuropsychological changes following STN DBS [41]. They found that passing the STN DBS lead through the head of the caudate increased the risk of decline in global cognition and working memory performance. In addition, this study also indicated that PD patients with a decline in cognition after STN DBS showed a different position of the active DBS electrodes in comparison to the one without a decline in the cognition. Thus, the finding from Witt et al together with the finding that the GPi target is more lateral to midline structures than the STN target[42] infers that the DBS lead trajectory required to target the GPi passes laterally or along a steeper angle that may theoretically spare the caudate area, which may lead to a lower risk of cognitive decline. However, to the best of our knowledge, no studies have reported on the relationship between the GPi DBS procedure itself and neurocognitive outcomes in PD patients. This inference needs further verification in subsequent studies. Secondly, it has been demonstrated that STN DBS is associated with a significant reduction in levodopa-equivalent dosages after DBS compared with GPi DBS[16,23,30]. Mounting evidence suggests that medication withdrawal following DBS in PD patients exacerbates neuropsychological symptoms and may be undesirable in some patients [43,44]. Furthermore, the potential role of medication reductions in the neurocognitive sequelae is supported by the findings of the Rothlind study, which indicated that the differences in Digit Symbol performance (which belongs to the domain of attention, working memory and processing speed) between the two groups were no longer significant after adjusting for the reduction in dopaminergic medication [28]. Further studies are needed to clarify these issues, and these studies may assist researchers in identifying the factors that underlie these differences.

In addition, our research found that STN DBS and GPi DBS showed similar depression scores after DBS, which was inconsistent with the previous report that showed greater improvement in depression from baseline after GPi DBS compared with STN DBS [23]. We considered that different assessment time-points account for this discrepancy. In both our research and the Liu et al study[23], the same three studies (Rothlind study, COMPARE trial and CSP 468 study) were included for analysis of depression. However, in our research, the follow-up periods were 15, 36, and 7 months after surgery in Rothlind study, CSP 468 study [29] and COMPARE trial[17], respectively. And, in Liu et al study, the follow-up periods were 6, 24, and 6 months after surgery in Rothlind study, CSP 468 study[16] and COMPARE trial[26], respectively, which indicating the longer periods of follow-up analyzed in our research. Previous meta-analysis [25] has demonstrated that the improvement of depression is significantly related with the follow-up time, and the effect of DBS on depression seems to wane in later assessments after initial improvement. More research is necessary to further explore the mechanisms underlying depression outcome following DBS.

There are some limitations to the present study. Chief among these limitations is the limited sample size. Although we have attempted to bring together all of the relevant RCTs in this meta-analysis, our analysis of primary and secondary outcomes remains limited by the small number of included patients and trials. Whereas the effects found in verbal fluency were based on total samples of 180 and 157 patients from all four of the include trials, some other effects, such as Digit span forward, were based on fewer than 20 individuals, and only one effect included in the trial reported the primary outcome (Table 2). Moreover, there may be loss of follow-up or withdraw in some studies, which leads to a decreased sample size at final analysis. It is probable that in the near future, the results of an ongoing randomized clinical trial (NCT01870518) will shed light on this issue. Furthermore, we should notice that the included four RCTs in our study have tested the PD patients at different time points after surgery. As described previously, the different periods of follow-up may influence the effects of DBS on the cognition and affectivity. Thus, it is hard for us to strictly differentiate whether the worsening of cognitive domains is the result of DBS surgery or the disease progression based on current evidence. Further studies with uniform reporting formats in the follow-up period may be of benefits to address this issue. In addition, as assessed using MDRS scores, the baseline global cognitive function in the four included trials were comparable between the two groups (Table 1). However, the results of the baseline neurocognitive tests in each included trials are more complicated. For example, the scores in some baseline neurocognitive tests were slightly worse in the STN DBS group than in the GPi DBS group in the CSP 468 study [16]. The COMPARE trial reported baseline task scores only for verbal fluency but not for other neurocognitive tests [17]. Baseline neurocognitive test scores were not compared between STN DBS and GPi DBS in the Rothlind study and were not reported in the NSTAPS study [21,28]. The results of future RCTs may be more convincing if balanced baseline parameters are established for all neurocognitive tests. Finally, individual patient data, such as age, levodopa-equivalent dosage, lead trajectory, and the final location of the implanted lead, are not generally included in the existing literature. These factors have been shown to affect the outcome and the size of effects. A future meta-analysis that is stratified by these potential outcome factors may be of benefit in identifying the factors that underlie cognitive morbidity and can be used to determine which PD patients are most likely to benefit from DBS treatment.

## Conclusions

A selective decline in frontal-subcortical cognitive functions is observed after STN DBS in comparison with GPi DBS, which should not be ignored in the target selection for DBS treatment in PD patients. In addition, compared to GPi DBS, STN DBS does not affect depression, anxiety, and quality of life.

## Supporting Information

S1 PRISMA Checklist PRISMA checklist. (DOC)

S1 Search Strategy Search strategy with details. (DOCX)

S1 Table Inclusion criteria of the four included trials. (DOCX)
