# Supplementary material for: Cognitive and Psychiatric Effects of STN versus GPi Deep Brain Stimulation in Parkinson's Disease: A Meta-Analysis of Randomized Controlled Trials
Source: PLoS One. 2016 Jun 1;11(6):e0156721. doi: 10.1371/journal.pone.0156721 (PMC4889151; doi:10.1371/journal.pone.0156721)
Supplement: S1 Search Strategy — (DOCX) [file pone.0156721.s002.docx]

**Details of Search Strategy**

**PUBMED**

((((((((((((((((((clinical trial[Publication Type]) OR (((((((((randomized[Text Word]) OR randomized[Text Word]) OR randomly[Text Word]) OR random order[Text Word]) OR random sequence[Text Word]) OR random allocation[Text Word]) OR randomly allocated[Text Word]) OR at random[Text Word]) OR controlled clinical trial*[Text Word]))) NOT ((((((("Models, Animal"[Mesh]) OR "Animals"[Mesh]) OR "Animal Experimentation"[Mesh]) OR "Animals, Laboratory"[Mesh]) OR "Disease Models, Animal"[Mesh])) NOT humans[MeSH Terms]))))))))))) AND ((((((((((((((((cognition[MeSH Terms]) OR neuropsychology[MeSH Terms]) OR psychiatry[MeSH Terms]) OR cognition[Title/Abstract]) OR cognitive[Title/Abstract]) OR neuropsychological[Title/Abstract]) OR neuropsychologic[Title/Abstract]) OR psychiatric[Title/Abstract]) OR psychiatry[Title/Abstract]) OR mood[Title/Abstract]) OR memory[Title/Abstract]) OR attention[Title/Abstract]) OR executive[Title/Abstract]) OR perceptual organisation[Title/Abstract]) OR verbal fluency[Title/Abstract]) OR processing speed[Title/Abstract])) AND (((((deep brain stimulation[MeSH Terms]) OR deep brain stimulation[Title/Abstract]) OR neurostimulation[Title/Abstract]) OR brain stimulation[Title/Abstract]) OR electrical stimulation[Title/Abstract])) AND (((parkinson's disease[MeSH Terms]) OR parkinson's disease[Title/Abstract]) OR parkinson disease[Title/Abstract])

**EMBASE**

randomised OR randomized OR randomly OR random AND order OR random AND sequence OR random AND allocation OR randomly AND allocated OR at AND random OR controlled AND clinical AND trial* OR 'clinical trial (topic)'/exp OR 'clinical trial (topic)' NOT ('animal model'/exp OR 'animal model' OR ('animal'/exp OR 'animal' AND ('experiment'/exp OR 'experiment')) OR 'animal'/exp OR 'animal' OR 'experimental animal'/exp OR 'experimental animal' NOT ('human'/exp OR 'human')) AND ('cognition':ab,ti OR 'neuropsychology':ab,ti OR 'psychiatry':ab,ti OR cognition OR cognitive OR neuropsychological OR neuropsychologic OR psychiatry OR psychiatric OR mood OR memory OR attention OR executive OR 'perceptual organisation' OR 'verbal fluency' OR 'processing speed') AND ('deep brain stimulation':ab,ti OR 'deep brain stimulation' OR 'neurostimulation' OR 'brain stimulation' OR 'electrical stimulation') AND ('parkinson disease':ab,ti OR 'parkinson disease' OR 'parkinsons disease')

**CENTRAL**

#1 MeSH descriptor: [Cognition] explode all trees

#2 MeSH descriptor: [Neuropsychology] explode all trees

#3 MeSH descriptor: [Psychiatry] explode all trees

#4 "cognition":ti,ab,kw or "cognitive":ti,ab,kw or "neuropsychologic":ti,ab,kw or "neuropsychological":ti,ab,kw or "psychiatric":ti,ab,kw (Word variations have been searched)

#5 "psychiatry":ti,ab,kw or "mood":ti,ab,kw or "memory":ti,ab,kw or "attention":ti,ab,kw or "executive":ti,ab,kw (Word variations have been searched)

#6 "perceptual organisation":ti,ab,kw or "verbal fluency":ti,ab,kw or processing speed:ti,ab,kw (Word variations have been searched)

#7 #1 or #2 or #3 or #4 or #5 or #6

#8 MeSH descriptor: [Deep Brain Stimulation] explode all trees

#9 "deep brain stimulation":ti,ab,kw or "neurostimulation":ti,ab,kw or brain stimulation:ti,ab,kw or "electrical stimulation":ti,ab,kw (Word variations have been searched)

#10 #8 or #9

#11 MeSH descriptor: [Parkinson Disease] explode all trees

#12 "Parkinson disease":ti,ab,kw or "Parkinson's disease":ti,ab,kw (Word variations have been searched)

#13 #11 or #12

#14 #7 and #10 and #13
