# Supplementary material for: Cognitive and Psychiatric Effects of STN versus GPi Deep Brain Stimulation in Parkinson's Disease: A Meta-Analysis of Randomized Controlled Trials
Source: PLoS One. 2016 Jun 1;11(6):e0156721. doi: 10.1371/journal.pone.0156721 (PMC4889151; doi:10.1371/journal.pone.0156721)
Supplement: S1 Table — (DOCX) [file pone.0156721.s003.docx]

|  | **Inclusion criteria** |
| --- | --- |
| **Rothlind et al, 2007** | 1. levodopa-responsive idiopathic PD producing disabling motor symptoms despite optimized pharmacotherapy 2. stable antiparkinsonian medication for at least one month prior to enrollment 3. moderate level of disability defined as Hoehn-Yahr Stage 3 or 4 and supported by UPDRS Motor Scale rating 4. no significant brain atrophy on brain MRI or CT 5. no major psychiatric disorder or dementia that would interfere with their ability to comply with follow-up for stimulator programming and assessment |
|  |  |
| **Okun et al, 2009 and Zahodne et al, 2009 (COMPARE Trial)** | 1. meet UK PD Brain Bank Criteria 2. be 30 to 75 years old, be right-handed 3. have an adequate response to levodopa(ie, an improvement of 30% on the UPDRS motor subscale in the “on” compared with the “off” medication state) 4. have disabling motor fluctuations or dyskinesia |
|  |  |
| **Follett et al, 2010 and Weaver et al, 2012 (CSP 468 study)** | 1. idiopathic Parkinson’s disease who were at least 21 years of age 2. stage 2 or higher on the basis of the Hoehn and Yahr disability scale while not receiving antiparkinsonian medication 3. had a response to levodopa 4. had persistent and disabling symptoms(e.g. motor fluctuations and dyskinesia) despite optimal medical therapy 5. had at least 3 hours per 24-hour period with poor motor function or symptom control 6. had been receiving medical therapy with no changes in the regimen for at least 1 month |
| **Odekerken et al, 2013 and Odekerken et al, 2015 (NSTAPS study)** | 1. patients aged 18 years or older who had idiopathic PD 2. despite optimum pharmacological treatment, at least one of the following symptoms: severe response ﬂuctuations, dyskinesia, painful dystonia or bradykinesia 3. did not have previous functional stereotactic neurosurgery 4. Hoehn-Yahr stage less than 5 at the best moment during the day, Mattis dementia rating scale score more than 120(out of 144) 5. did not have active psychosis, or contraindications for the neurosurgical procedure |

**Supplementary Table: Inclusion criteria in each trial.**
